# Plasmonic nanostructure design and characterization via Deep Learning

**DOI:** 10.1038/s41377-018-0060-7

**Published:** 2018-09-05

**Authors:** Itzik Malkiel, Michael Mrejen, Achiya Nagler, Uri Arieli, Lior Wolf, Haim Suchowski

**Affiliations:** 1 School of Computer Science, Faculty of Exact Sciences, Tel Aviv University, Tel Aviv, 69978 Israel; 2 School of Physics and Astronomy, Faculty of Exact Sciences, Tel Aviv University, Tel Aviv, 69978 Israel

## Abstract

Nanophotonics, the field that merges photonics and nanotechnology, has in recent years revolutionized the field of optics by enabling the manipulation of light–matter interactions with subwavelength structures. However, despite the many advances in this field, the design, fabrication and characterization has remained widely an iterative process in which the designer guesses a structure and solves the Maxwell’s equations for it. In contrast, the inverse problem, i.e., obtaining a geometry for a desired electromagnetic response, remains a challenging and time-consuming task within the boundaries of very specific assumptions. Here, we experimentally demonstrate that a novel Deep Neural Network trained with thousands of synthetic experiments is not only able to retrieve subwavelength dimensions from solely far-field measurements but is also capable of directly addressing the inverse problem. Our approach allows the rapid design and characterization of metasurface-based optical elements as well as optimal nanostructures for targeted chemicals and biomolecules, which are critical for sensing, imaging and integrated spectroscopy applications.

## Optics: Controlling light at the nanoscale

Scientists have used the power of computing to design tiny structures capable of controlling light at the nanoscale, opening the door for new applications in sensing, imaging and spectroscopy. The emerging field of nano-photonics, which enables the manipulation of light-matter interactions using nanostructures, has revolutionized the field of optics. However, designing a nanostructure that produces a desired optical response is very challenging. Rising to the challenge, Haim Suchowski and colleagues from Tel Aviv University in Israel have developed an innovative technique that uses Deep Neural Networks to model the complex relationships between light-matter interactions, allowing them to characterise nanostructures based on their far-field optical responses. Their approach provides a rapid and efficient method for the designing the optical responses of nanostructures, and could be used in a range of applications, including sensing and imaging.

## Introduction

In recent decades, many breakthroughs in optics have led to unprecedented imaging capabilities beyond the diffraction limit, with applications in biology and nanotechnology. In this context, nanophotonics has revolutionized the field of optics in recent years by enabling the manipulation of light–matter interactions with subwavelength structures^1–3^. However, despite the many advances in this field, its impact and penetration in our daily life has been hindered by a convoluted and iterative process, cycling through modeling, nanofabrication and nanocharacterization. The fundamental reason starts with the fact that the prediction of the optical response is very time consuming and requires solving Maxwell's equations with dedicated numerical packages^4,5^ http://www.lumerical.com/tcad-products/fdtd/. Next, more significantly, the inverse problem, i.e., designing a nanostructure with an on-demand optical response, is currently a prohibitive task even with the most advanced numerical tools due to the high nonlinearity of the problem^6,7^. In parallel, for many years, computer science has been harnessed to address challenging tasks in nanophotonic imaging, design and characterization. In general, the approaches were either to target enhancing/resolving imaging and characterization beyond the diffraction limit (super-resolution techniques such as PALM and STORM techniques and more https://www.nobelprize.org/nobel_prizes/chemistry/laureates/2014/advanced-chemistryprize2014.pdf ^8–10^) or to assist with the design process of nanophotonic devices^11–17^. However, to date, very few computational techniques are capable of addressing both aspects in an integrated manner for nanoplasmonics. In this context, Deep Learning (DL) has emerged in recent years as a very powerful computational method that has achieved state-of-the-art results in various tasks, including computer vision^18^, speech recognition^19^, natural language processing^20^, face recognition and other applications^21^. Inspired by the layered and hierarchical deep architecture of the human brain, DL uses multiple layers of nonlinear transformation to model high-level abstraction in data. DL has also been successfully employed in research areas beyond computer science, such as in particle physics^22^, ultra cold science^23^, condensed matter^24^, chemical physics^25^ and conventional microscopy^26,27.^

Here we present an integrated DL approach and show how deep neural networks (DNNs) can streamline the design process and provide a unique, robust, time-efficient and accurate characterization capability for complex nanostructures based on their far-field optical responses. The complexity of the DNN can address the high level of nonlinearity of the inference tasks by creating a model that holds bidirectional knowledge. While it is common practice in DL to separate different problems and to train multiple separate networks for each problem, We show that our approach of training a *bidirectional* network that goes from the optical response spectrum to the nanoparticle geometry and back is significantly more effective for both the design and characterization tasks. Furthermore, we show that this DL approach not only can predict the spectral response of nanostructures with high accuracy but also can address the inverse problem and provide a single nanostructure’s design, geometry and dimension, for a targeted optical response for both polarizations.

This DL approach provides a method for direct on-demand engineering of plasmonic structures and metasurfaces for applications in sensing, targeted therapy and more. Moreover, the predictive capability of the DL model also holds great promise for multivariate characterization of nanostructures beyond the diffraction limit.

## Results

**Fig. 1:**
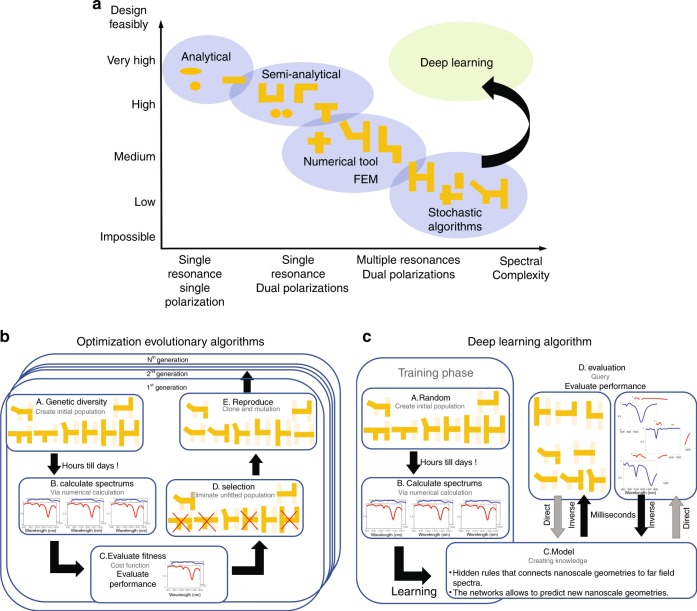
Comparison of the different computational approaches to plasmonic nanostructure design **a** To date, the approaches enabled by the computational tools available are efficient only for ‘direct’ modeling, i.e., predicting the optical response in both polarizations of a nanostructure based on its geometry, constituent and surrounding media. However, the ‘inverse’ problem, where the tool outputs a nanostructure for an input desired optical response, is much more relevant from a designer’s point of view and is currently inefficient and time consuming. The more complex the desired optical response, the more unattainable the solution of the inverse problem. **b**, **c** Optimization algorithm vs. DL in nanophotonics and the ‘inverse’ problem. **b** The Genetic Algorithm applied to the field of predicting nanophotonic geometries and their responses. The typical Genetic Algorithm contains the following five phases: (A) Create the genetic diversity that serves as an initial population of various nanostructures. (B) Calculate the spectrum for each configuration, which is a time-consuming task for complex nanostructures. (C) Evaluate the fitness. (D) Eliminate the least fit nanostructures (the selection phase). The population is examined to find a set number of top scores, and any solution with a poorer fitness score is eliminated from the population. (E) Clone and mutate the most fitted structures (the reproduction phase). The frequencies and types (deletion, addition, variations) of the mutations can vary. The output is the best fit sample found throughout the generations. GA is a classic example of an optimization algorithm, where a multidimensional space is searched for each and every design task. **c** Building a Deep Learning model typically involves the following four phases: (A) Create a data set with diverse experiments. (B) Calculate the spectrum for each configuration. This step is a time-consuming task for complex nanostructures. (C) Learn an optimized model using the data set. (D) Query the trained model with new experiments. To predict beyond the training data, the network must learn the inner set of rules of the nanostructure optical response phenomena. This approach can therefore be considered to be an optimization that is performed once for all future cases. Once the inner set of rules is learned, the query phase is extremely fast and accurate

To demonstrate the paradigm shift that is enabled by our Deep Learning approach, we consider the interaction of light with subwavelength structures such as plasmonic nanostructures, metamaterials and composite layered metallic nanostructures embedded in dielectrics, which allow control of the properties of the outgoing light^28^. Predicting the far-field optical response for a defined nanostructure geometry and composition involves solving the full set of Maxwell equations at each location in space and for each wavelength. However, whereas the far-field spectrum is directly connected to the nanostructure geometry, the solvability of the ‘inverse’ problem, i.e., inferring the nanoscale geometry from a measured or desired far-field spectrum, depends to a large extent on the complexity of the system of interest (Fig. 1a).

For a simple nanostructure, which exhibits single resonance peaks in each polarization, one can solve it semi-analytically or in an intuitive manner^29^; however, for a general spectral response associated with more complex geometries, no analytical solution is known, and time-consuming numerical methods such as Finite Element Method (FEM) or Finite Difference Time Domain (FDTD) method must be used. Further optimization methods such as shallow neural networks, evolutionary algorithms and linear regression^11,13,30,31^ have gained some success in solving the inverse problem task. However, current techniques are still limited in accuracy and practical feasibility and fall short in the modeling of nonlinear problems with high complexity of the underlying physical processes. To date, none of these approaches can efficiently address the inverse problem, and it still takes many cycles of trial and error of modeling and characterization to predict or design a nanostructure for a desired or measured far-field optical spectral response^31^.

We emphasize that the Deep Learning approach presented here is fundamentally different from evolutionary approaches since, for every single design task, the evolutionary approaches search the parameter space over dozens (sometimes hundreds) of generations, with each generation encompassing dozens/hundreds of individuals (Fig. 1b). For this reason, the individuals should be simple enough to enable their electromagnetic response to be analytically solved; otherwise, the optimization task takes a prohibitive amount of time, which limits the usefulness of such an approach. Our approach is radically different (Fig. 1c). We train our DNN on a set encompassing structures that are not trivial and for which responses must be calculated using time-consuming numerical approaches. However, once the data set is created and learned, this task is nonrecurring and each design task requires only a query of the DNN that takes not more than a few milliseconds.

**Fig. 2:**
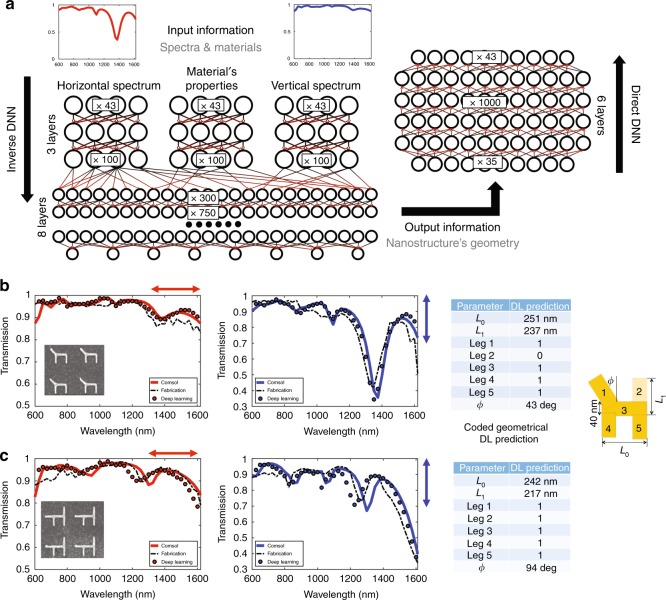
Deep-learning network architecture and results. **a** The deep networks have a cascaded structure of many layers of nonlinear processing units, where each layer uses the output from the previous layer as input. The training of our bidirectional network consists of two phases. We first train the inverse network to predict the geometry based on the transmission spectrum. In the second phase, we train the direct network on top of the first network. The inverse network receives, as input parameters, two spectra and material properties, and for each experiment, it learns the corresponding geometry, material properties and resonances of the unknown geometry. For more detail, see the Supplemental document. Once this DNN is trained, the nanostructure's geometry is retrieved based on the measured/desired transmission spectrum by querying the inverse network. **b**, **c** Deep Learning retrieval results for two different gold nanostructures that we fabricated are depicted. The measured spectrum is depicted in a red (blue) dotted line for the horizontal (vertical) input polarization. The DL predicted geometry is represented by the different lengths in the table. The DL spectrum based on the predicted geometry is depicted as full circles. The results of the COMSOL simulations based on the DL predicted geometry are represented as full lines. For all of the nanostructures, the gold thickness is maintained at 40 nm. Further information on the Deep Learning network and a comparison of simple structures can be found in the Supplemental Document

To illustrate our approach, we design a novel deep network that uses a fully connected neural network. We introduce a bidirectional deep neural network architecture that is composed of two networks (Fig. 2a), where the first is a Geometry-predicting-network (GPN) that predicts a geometry based on the spectra (the inverse path) and the second is a Spectrum-predicting-network (SPN) that predicts the spectra based on the nanoparticle geometry (the direct path). The geometry predicted by the GPN is fed into the SPN which, in turn, predicts the spectrum. We thus solve the harder inverse problem first, i.e., predicting the geometry based on two spectra for both polarizations, and then, using the predicted geometry, we match the recovered spectrum with the original one (see Supplemental Document for further information). It is worthwhile to note that the training of such a bidirectional network requires a dedicated learning procedure, since the input to the SPN is a predicted geometry rather than the actual geometry (see Supplemental Document for more information). Furthermore, we also observe a significant gain from training one network on *all* the training sets rather than the alternative of training multiple separate networks. It is crucial to stress that the learning phase in the DNN is a nonrecurring effort, which means that once the data set is learned, the query phase is quasi instantaneous. This approach is a clear departure from evolutionary methods in which for every query, the whole parameter space is searched for optimization.

To train our DNN, we created a large set of synthetic data using COMSOL Multiphysics^4^. The data contain more than 15,000 experiments, where each experiment is composed of a plasmonic nanostructure with a defined geometry, its metal properties, the host's permittivity and the optical response spectrum for both horizontal and vertical polarizations of the incoming field. While we maintain a constant thickness of the nanoparticle, the thickness can of course influence the transmission spectra (blueshift and resonance strength). This variable can be added as a parameter to the learning data set and allow refined predictions. In our proof of concept, we choose a nanostructure geometry represented by a general "H" form that can be easily fabricated using top-down approaches, where each of the outer edges can vary in length and angle or can be omitted (Fig. 2). Such variable geometry is sufficiently complex to span a wide variety of optical response spectra for both polarizations. We then feed the DNN with these synthetic optical experiments and let it learn the multivariate relationship between the spectra and all of the aforementioned geometric parameters. During this training process, the prediction provided by the DNN on a set of synthetic experiments is compared to the COMSOL solutions, and the network weights are optimized to minimize the discrepancy. A set of similarly created samples, unseen during training, is used to evaluate the network’s performance.

We then demonstrate our DNN’s ability to accurately predict the fabricated nanostructures’ parameters beyond simulations, by fabricating a set of different geometries that encompass some geometries that the network has never seen before. Those geometries were fabricated with gold on ITO covered glass (see “Methods” section). We measured the transmission spectra on a home-built reflection-transmission setup (see “Methods” section).

We fed these *measured* spectra into our trained DNN and obtained excellent agreement between the retrieved dimensions and those actually measured by the SEM (Fig. 2). These excellent predictions were obtained once the DNN was trained with an additional training set of 1500 simulated geometries (each of the geometries was considered under the two polarization illuminations), for which the network was able to learn the different geometries’ responses in the presence of the *measured* dispersion of the indium tin oxide layer (ITO). We emphasize that our DNN allows the retrieval of geometrical dimensions and optical properties of a subwavelength geometry that reproduce its far-field spectra from the family of subwavelength H-geometries.

This finding is, to our knowledge, an impressive capability of multivariate parameter retrieval. We note that this achievement is enabled by the unique bidirectional architecture and the simultaneous learning process between the GPN and SPN, which leads to co-adaptation between the networks. Compared to the simultaneous bidirectional training, we observed that the performance of the two separately trained GPN and SPN is significantly inferior.

The bidirectionality, where the output of the inverse network serves as an input to the direct network and is used to predict the two spectrums of the predicted geometry, constitutes a unique feature of our network and is therefore further investigated. As an example, we demonstrate the bidirectionality advantage in the case of the dispersive ITO. This advantage is apparent from the Mean Squared Error (MSE) achieved on the error function in both approaches, i.e., bidirectional versus composite direct (SPN) and inverse (GPN) networks (more information can be found in Supplemental Document). The bidirectional network exhibits a significantly lower MSE of 0.16 compared to the MSE achieved with the composite approach (MSE = 0.37).

**Fig. 3:**
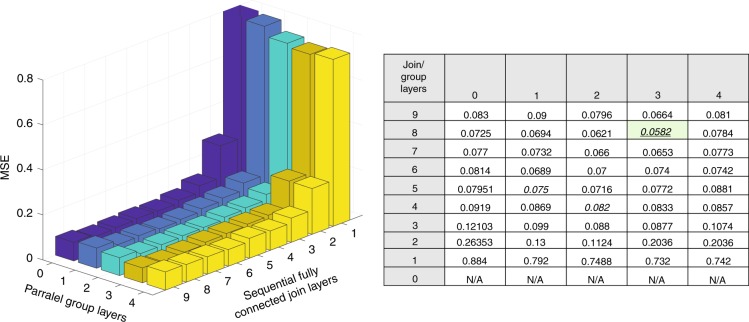
Analysis of the depth and parallelism of the network. The performances of a trained GPN with different architectures are presented. For each number of parallel layers, we change the number of fully connected layers, namely, the Join-layers depth. In this case, a deeper DNN leads to better accuracy. However, there is a certain depth, in our case eight layers, in which the addition of extra layers does not improve the results or leads to the vanishing gradient phenomenon. The importance of the parallel group layer part of the GPN is also demonstrated. Adding parallel layers forces the network to represent each one of the three data groups separately and only then join them together into one large inner representation. Using parallel group layers improves the results, but the number of layers should be chosen carefully. For comparison, a classical shallow network that has only one hidden layer completely fails at the prediction task, with an MSE of over 0.7

To gain insight on the effect of the network’s depth on the prediction performance, we conduct an extensive comparison between different network architectures. We show that different network depths have a dramatic effect on the results. We vary the number of fully connected layers at the second part of the inverse network, and by comparing the results to one another, we see a significant effect on the accuracy of the prediction, as seen in Fig. 3. We find that the best inverse network architecture for our case is three parallel group layers followed by eight sequential fully connected join layers. Interestingly, we observe a significant gain in accuracy when using eight join layers compared to five or seven layers in the sequential part of the network. The benefit of such a deep network is directly derived from the complexity and nonlinearity of the underlying physical process. We observe a significant gain from training one network on all of the training set over the alternative of training multiple separate networks. While this finding can be attributed to the so-called transfer of knowledge^32^, where knowledge learned from one problem is transferred to another, we are not aware of other instances in which it is that crucial to train one single generalist network instead of applying a divide-and-conquer strategy with multiple specialist networks.

To test the boundaries of the DDN retrieval, we check the performance of our DNN to address radically unseen cases such as no nanostructures being present in the queried spectra (meaning that the spectra will be approximately flat with 100% transmission in both axes). The DNN is presented with the horizontal and vertical input polarization spectra.

**Fig. 4:**
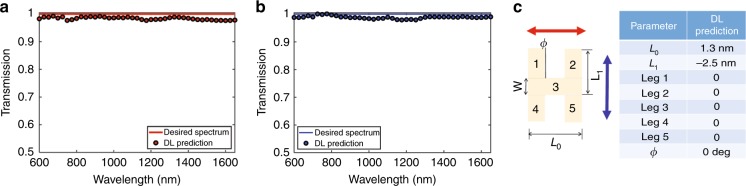
Deep Learning retrieval of “no nanostructure”, a boundary case for which the DNN has not seen anything close during the training phase. **a** Horizontal and (**b**) perpendicular polarization spectra. **c** The network could retrieve the output of all of the “Leg” flags as 0

We observe (Fig. 4) that out of all of the infinite possibilities (the returned lengths could have, for example, blown), the network output matched the reality without previously seeing this geometry. This finding shows that the DNN is not simply “interpolating”, as there is nothing even close to the “none” case in the training set; in fact, the DNN performed generalization. Additionally, it is worthwhile to mention that even in the angle parameters and the two lengths, the output of the network was at the appropriate scale.

**Fig. 5:**
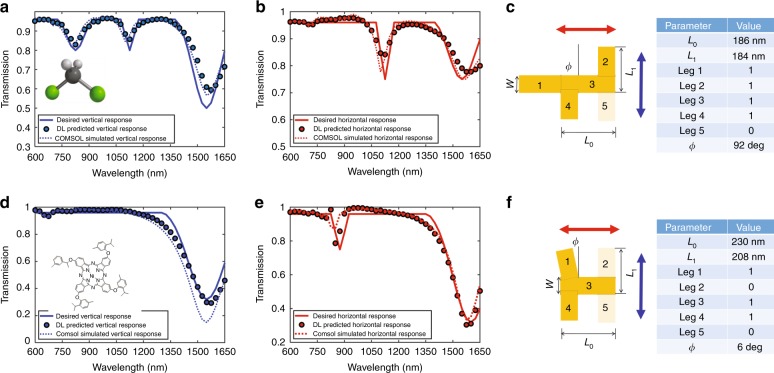
Prediction of the nanostructure’s geometry for chemical sensing. **a**–**c** DNN-based design of a gold plasmonic structure targeted to the organic molecule dichloromethane with different spectral polarization responses (**a**, **b**) on one polarization axis. It has two resonances on 1150 nm and a broad resonance between 1400 and 1600 nm, whereas on the orthogonal polarization axis, it has three resonances, at approximately 820 nm (matching a Ti:Sapphire femtosecond laser excitation for a pump-probe experiment), 1064 nm and 1550 nm. **c** Configuration and dimensions of the plasmonic structure found by the DNN. **d**, **e** The targeted molecule is asymmetrical phthalocyanine dimer 1a, a synthetic molecule that has more complex polarization characteristics and has potential applications due to its charge transfer properties. **f** Configuration and dimensions of the plasmonic structure found by the DNN. In both geometries, after prediction of the geometry, COMSOL simulations were performed, which showed excellent agreement with the desired spectra. This design approach can be extended to other molecules for biology, chemistry or material sciences

Next, we have examined the strength of the inverse predictive approach for sensing applications in which plasmonic nanostructures are used to enhance the light–matter interactions with various chemicals and biomolecules. Organic compounds typically exhibit pronounced resonances across the spectrum from ultraviolet to mid-infrared. We show that our trained DNN allows us to find the nanostructure configuration to best interact with a given molecule with target multiple resonances in the two polarizations. More specifically, we wish to design a nanostructure that is targeted at enhancing the interaction with dichloromethane, an important chemical used in industrial processes. This organic compound exhibits one resonance at ~1150 nm and another at approximately 1400–1500 nm https://commons.wikimedia.org/wiki/File:Dichloromethane_near_IR_spectrum.png#/media/File:Dichloromethane_near_IR_spectrum.png. Our design goal is to achieve a nanostructure that will resonate in an aqueous solution (at both wavelengths for one polarization and with completely different resonances at the orthogonal polarization, at ~820 nm (matching a Ti:Sapphire femtosecond laser excitation for a pump-probe experiment), 1064 nm and 1550 nm (Fig. 5a, b). In the existing design process, this task would require to iterate through different designs using the standard FEM or FDTD simulation tools, a process that can be extremely time consuming. The DNN’s inverse solution yields, in a few seconds, the parameters shown in Fig. 5c). We also applied this design approach to the asymmetrical phthalocyanine dimer 1a, a synthetic molecule that has more complex polarization characteristics (Fig. 5d, e) and has potential applications due to its charge transfer properties^33^. The DNN inverse design for this targeted molecule and polarizations results in the configuration shown in Fig. 5f. This finding demonstrates the capability of our DNN to address various targeted resonances in different polarizations and emphasizes that this approach can be extended to other molecules for sensing in biology, chemistry and material science.

## Discussion

In conclusion, we introduce a novel deep-learning approach for predicting the geometries of nanostructures based solely on their far-field responses. We have designed, trained and tested the proposed scheme, showing a very accurate prediction of the geometry of a complex nanostructure. This approach could be extended to other physical and optical parameters of the host materials and compounds. The approach also effectively addresses the currently inaccessible inverse problem of designing a geometry for a desired optical response spectrum and significantly speeds up the direct spectrum prediction of such subwavelength structures. This approach allows for the on-demand design of optical responses of nanostructures and metasurfaces for many applications, such as sensing, imaging and more.

## Materials and Methods

### Preparation

ITO covered glass (Sigma Aldrich) were covered with PMMA-A4 polymer and spin-coated for one minute at 7,000 RPM. The electron beam (Raith150) used was a 10 kV beam, aperture 6 mm WD, and a dose was deposited in single-pixel lines. Samples were then developed in MIBK/IPA (1:3) for 1 min and rinsed in isopropanol for 20 s. A concentration of 40 nm of gold was then evaporated on the sample with an E-Beam Evaporator (VST evaporator). Lift-off was performed with acetone and followed with a final wash in isopropanol.

### Sample characterization

Sample sizes were verified using an electron microscope and were optically characterized using an OSL2 Broadband Halogen Fiber Optic Illuminator (Thorlabs) light-source and LPNIR050 (Thorlabs) broad band polarizer. Transmitted light was filtered in an imaging plane by an iris such that only light that passed through the sample was collected and then analyzed by an AQ6370D (Yokogawa) spectrometer.

### COMSOL simulation

We performed finite element method (FEM) simulations using the 'Electromagnetic Waves, Frequency Domain' module of the COMSOL 4.3b commercial software. For consistency, the edges were made using fillets with a constant radius of 15 nm. We have considered geometries based on a five edges shape of 'H' while varying an angle of one of the edges, the existing edges and the edges’ lengths.

The nanostructure is simulated in a homogeneous dielectric medium with a chosen real effective-permittivity. For preventing reflections from the far planes, PMLs with a depth of the maximum wavelength were placed on both far ends of the homogeneous medium in the propagation direction of the radiating field.

For the data set predicting the fabrications, the nanostructure made of Gold was modeled with a wavelength-dependent homogeneous medium permittivity, and where the ITO permittivity is wavelength dependent, such that its imaginary part can be neglected in the measured spectrum range. It has been shown that changes in the thickness of a Titanium adhesion layer higher than 40% of the nanostructures’ height do not affect the plasmon resonance. Furthermore, for an Au nanoparticle with a diameter of 10 nm and a graphene layer, the LSPR shifting saturates when the distance is >20 nm.

A prediction for a similar behavior of the ITO layer is assumed. In our case, the ITO thickness is ~100 nm, which is approximately 250% of the nanostructure thickness of ~40 nm.

## Electronic supplementary material

Supplementary Material for “Plasmonic nanostructure design and characterization via Deep Learning”

## Data Availability

The open source code for the DNN presented in this work can be found at the following URL https://github.com/ItzikMalkiel/DeepNanoDesign
