# Supplementary material for: Plasmonic nanostructure design and characterization via Deep Learning
Source: Light Sci Appl. 2018 Sep 5;7:60. doi: 10.1038/s41377-018-0060-7 (PMC6123479; doi:10.1038/s41377-018-0060-7)
Supplement: Supplementary file 1 — Supplementary Material for “Plasmonic nanostructure design and characterization via Deep Learning” [file 41377_2018_60_MOESM2_ESM.docx]

Supplementary Material for

“Plasmonic nanostructure design and characterization via Deep Learning”

Itzik Malkiel^1,§^, Michael Mrejen^2,§^, Achiya Nagler^2,§^, Uri Arieli^2^, Lior Wolf ^1^ and Haim Suchowski^2,*^

^1^School of Computer Science, Faculty of Exact Sciences, Tel Aviv University, Tel Aviv 69978, Israel

^2^School of Physics and Astronomy, Faculty of Exact Sciences, Tel Aviv University, Tel Aviv 69978, Israel

**^§^**These authors contributed equally to this work

*Corresponding author: <mailto:haimsu@post.tau.ac.il>

^§^These authors contributed equally to this work

1. Further information of Deep Learning network
2. Architecture

The bi-directional deep neural network architecture is composed of two networks. The first network termed GPN is responsible for predicting geometry, and the second network ("SPN") predicts the spectrum of the predicted geometry. Both networks consist of fully connected layers that have rectified linear units (ReLUs, with activation function $max(0, x)$). The input to the first network consists of three groups of data:

1. Desired spectrum for X polarization, represented as a vector of 43 samples in the range of 600 nm – 1650 nm with spaces of 25 nm.

2. Desired spectrum for Y polarization, represented as a vector of 43 samples in the range of 600 nm – 1650 nm with spaces of 25 nm.

3. The materials’ properties, represented as a vector of 25 parameters.

The first network, GPN, uses a parallel architecture of three fully connected layers, where each layer receives an input from only one group out of the three above, i.e. each group of data is being fed into a different layer, which causes us to name them “group layers” (See visualization in Figure 2). Each group layer consists of 3 fully connected layers of 100 neurons each. The neurons of the three group layers are then concatenated together to one “join layer” that is followed by another seven fully connected joint layers, each composed of 750 neurons. The last layer of these eight joint layers composed of eight neurons that encode the predicted geometry.

SPN, the second network is composed of eight fully connected layers. The first layer receives as an input the eight output values of the first network, the materials’ properties and a flag that indicates the polarization. Every inner layer composed of 1000 neurons that are fully connected to the next layer. The last layer of the network consists of 43 neurons where each one of them represents the Y value of the transmission graph in predefined and fixed wavelength values (in this paper, we focused on 43 wavelength data points in the range 600 nm - 1650 nm).

1. Training

The networks are trained to minimize the mean squared error (MSE) between the predicted spectra and geometry to their ground truths.  These spectra and geometry are vectors where we first normalized, each value separately, to have a zero mean and a standard deviation of 1. This is done in order to reduce the influence of parameters with relatively big values compared to parameters with smaller values such as the transmission ratio (which is a number between 0 and 1). Adadelta (a gradient descent optimization algorithm), is used as an optimization method, and seems very helpful in reducing the sensitivity of the learning process to the exact learning rate used. A batch size of 64 samples is used throughout our experiments, since it seemed to lead to better performance in preliminary experiments.

As mentioned above, the output vector of SPN (the second network) is used to predict both polarizations, based on a binary flag that selects the desired one. We, therefore, train the second network with a batch size that is twice the batch size of the first network, using each training sample twice – once for each polarization direction. About 3000 epochs are performed in total until we receive the most optimized network with the best accuracy. The training takes around two hours in order to get the best results and approximately a half hour in order to get moderate results.

In order to train both GPN and SPN to maximize both performances, we use a simultaneous training process. As part of the training process, we iterate over the data, split it to batches and forward each batch through the GPN to predict geometry and then we forward the predicted geometry through the SPN. Next, we calculate the gradients by comparing the predictions to the ground truth and we perform the backward step. The backward is being performed on the SPN for every step, but in order to stabilize both networks, and in order to let the SPN learn the changes of the GPN, we don’t perform backward on the GPN every epoch. We backpropagate through GPN only once every K=100 iterations in order to allow the SPN sufficient time to adapt to the GPN.

1. Dataset

The dataset used consists of 3,000 simulated samples in the case of wavelength dependent ITO adhesion layer, each sampled according to 12 different nanostructure geometries. Each class of geometry has different edge length/angle/material properties. The experiments were composed of four different materials, and each experiment has two graphs of transmission per wavelength, one for each polarization. The wavelengths are fixed in the range of 600 nm to 1650 nm with a step size of 25 nm and the transmission is a value between 0 to 1 indicating the percentage of light that was transmitted through the nanostructure. A total of 80% of the samples are used for training, 5% for validation, and the remaining samples for testing. In this dataset the substrate is fully taken into account where we model the ITO adhesion layer that has a strong wavelength dependence around 1400 nm.

In addition, we generated a dataset of 15,000 simulated samples for the constant host permittivity of the same H family with the same properties, as described above, except to the host material. Each experiment of this data contains a host material represented by its permittivity value. The generated synthetic experiments composed of host material permittivity values that vary between 1 to 3, where 1 is air, (1.33)2 is water, (1.5)2 is Silica. Etc. The dataset was split to train, test and validation according to the host permittivity value, where all the experiments with host permittivity in the range [1.1…1.5] were in the test/validation and all the rest were used during the training process.

1. Bidirectional Neural Networks

When thinking about the SPN as a mapping of geometry to a spectrum; the GPN would be a mapping from a spectrum to geometry. While it is possible to train two networks individually, composing them together only post-training would cause the design process to become unstable: Given a pair of desired spectrums, the GPN would provide a geometry and the SPN would provide its spectrums. However, for two separate networks, there is no guarantee that the two spectrums predicted using the SPN applied to the predicted geometry would closely match the original spectrums. In other words, it is likely that there would be some drift when going back and forth. Since during the design process one is likely to adjust both the geometry and the shape of the spectrum to account for various constraints, this drifting will hinder the effectiveness of the process.

Instead, we propose to train one network that contains both SPN and GPN in order to optimize them together so they will co-adapt to each other. We call this process co-adaptation of networks. We solve the (harder) inverse problem first using the GPN, and then, using the predicted geometry, make sure that the recovered spectrum matches the original one using SPN. Training this type of a network requires a dedicated procedure since the input to the second half of the network is a predicted geometry and not the true geometry that is known to produce the spectrum. During the training procedure, we perform one forward pass on the GPN, then we clone the output to create two queries for each experiment - one for each polarization. These two queries are different in the polarization flag, indicating the relevant polarization. Since we use batch learning, we perform it on the entire batch and create a double sized batch for the SPN. We then perform forward on the SPN, by feeding it with the GPN output. At the end, we backward the two networks, calculate a loss for each one, sum the loss and send it to the optimizer with the gradients of both networks. We have also noticed a few training tricks that led to better results/time to converge. The first is doing this backward on the SPN only after a predefined amount of epochs, when the GPN becomes stable with moderate results. We also noticed that with each epoch, performing a few extra epochs without doing backward on the GPN leads to better results - i.e. we give the SPN more than one epoch to adjust to each GPN hypothesis during the training.

Loss Function

The loss function that we use is:

$$Loss = GPN\_MSE(predictedGeometry, groundTruthGeometry) + SPNx\_MSE(predictedSpetrumX, groundTruthSpectrumX) + SPNy\_MSE(predictedSpetrumY, groundTruthSpectrumY)$$

Where $\mathrm{predictedGeometry}$ is the ouput of the GPN and $\mathrm{groundTruthSpectrumX}$ and $\mathrm{PredictedSpetrumY}$are both the output of the SPN. See Fig. S1 and S2.


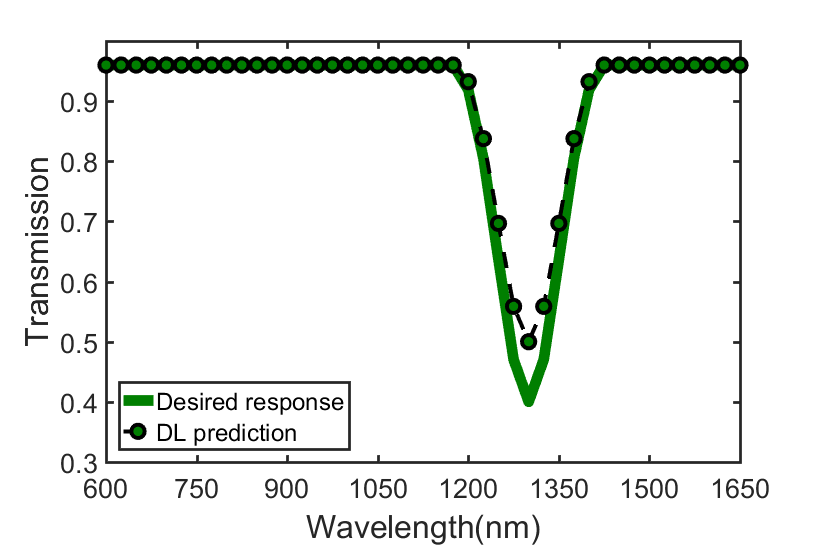


Fig. S1. Example of a good spectrum prediction, where the SPN MSE is low with MSE = 0.08 (i.e. the DL prediction is accurate).


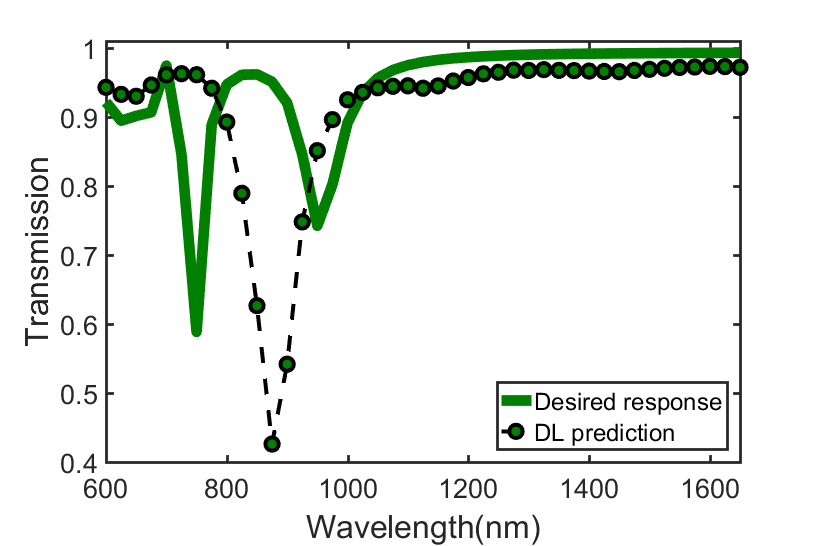


Fig. S2. Example of a spectrum prediction with a high SPN MSE with value of MSE = 1.71 (i.e. the DL prediction is very poor).

1. Assumptions

1) All the geometries are of H-form shaped and with 40 nm width and 40 nm thickness.

2) In order to design a geometry, the designer will provide both spectrum X and both spectrum Y of the wanted geometry.

3) The input spectrums are in the range of [0,1].

4) The experiments may include different dielectric value or an ITO adhesion layer.

5) The ranges of the geometry variables can vary, during the training process we automatically normalize each feature separately.

6) The network can't create design for any spectrums. The H family is spanning a space of potential spectrums, where not every spectrum can be modeled by an H geometry. There are spectrums that the network doesn't know to design geometry that suites them.

7) The transmission regressed using the last layer of the spectrum-prediction-network, where each neuron of the last layer corresponds to a-"y" at a fixed "x" value. For example, the first neuron output the value of the spectrum at wavelength 600 nm. The second neuron output the value of the spectrum at wavelength 625 nm and so on.

8) For the choice of the loss function, we kept in mind that its primary goal is to indicate the penalty for an incorrect prediction. Whereas there are several options for classification problems (Hinge loss, logistic loss etc.), when it comes to optimization and regression the loss function has to be globally continuous and differentiable. Therefore, the squared loss or MSE is very widespread in machine learning and in our case has proved to be very efficient.

We would like to emphasize that the description of the neural network itself is completely accessible to any researcher in the field of Machine Learning and that the learning part should be completely reproducible. Moreover, we add all the information of the Deep NNs that we worked with in this manuscript in Supplementary Materials, which contains also details of how we assemble those and the different parameters. We will also publish the source code and the learned weights of the networks online for the general use of the community.

1. Retrieval task of simple geometries

In order to test the generalization capability of the networks, we have performed the retrieval task of simple geometries from the H-form family - nanobar, L-shape and Split-ring-resonators (U-shapes). As discussed in the manuscript, our bi-directional deep neural network can predict very rapidly any nanostructure geometry from the H-form family. However, it was not trained on these simplified cases, with the exception of a few nanobars. Success of these cases would indicate that the networks have learned to generalize far beyond their training set.

The DNN was presented with measured spectra of samples (A) nanobars ,(B) “L”-shape and (C) “U-Shape” for which SEM measurements have been taken (“fabrication” column). We run COMSOL simulation with the DNN predicted set of measurements and present all those spectra for comparison. In Figure S3, we show the results of such retrieval for (A) nanobar (only one horizontal wire) (B) L-shaped (C) Split Ring Resonator (SRR). In all of the cases there is an excellent agreement between the DNN prediction, the actual measurement and the COMSOL simulation. We would like to emphasize that the generalization capability of our method, as demonstrated in the figure, is quite remarkable since only a couple of nanobars, several of L shapes and SRRs were included in the training set samples but only in epsilon host value of 1.0 (none of which are the predicted geometries).

­­


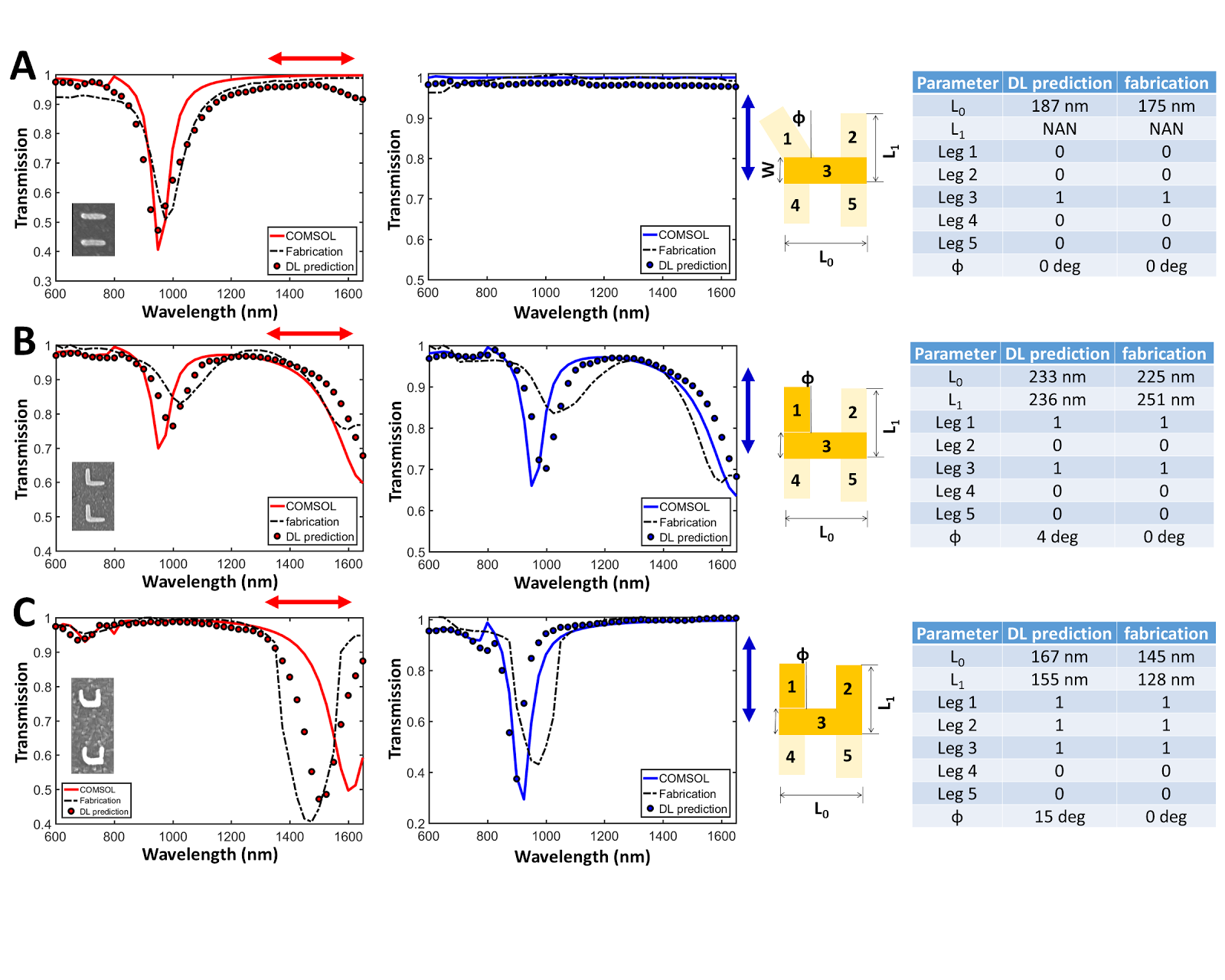


**(a)**

**(b)**

**(c)**

Fig. S3. DNN Retrieval for (a) nanobar (b) L-shaped (c) Split Ring Resonator (SRR). The DNN was presented with *measured* spectra of samples (a),(b),(c) (none of which has been seen by the DNN) for which SEM measurements have been taken (“fabrication” column). We run COMSOL simulation with the DNN predicted set of measurements and present all those spectra for comparison. In all of the cases there’s is an excellent agreement between the DNN prediction, the actual measurement and the COMSOL simulation.

1. The Learning Boundaries - retrieval task of none geometry and multiple geometries in a single unit cell

We have decided to check the performance of our DNN to deal with radically unseen cases such as (i) no nanostructures (meaning the spectra will be approximately flat 100% transmission in both axes) and (ii) several cases of more than one geometry (such as two nanobars that are not connected from the same H-form family). In all cases the DNN is presented with the horizontal and vertical input polarization spectra. First, COMSOL simulations were performed on the geometry that we wished to examine, and then the optical spectra of the two polarizations was fed as an input to our DNN.

We queried the DNN with spectra from structures that are lacking the connecting element (element #3 as seen in the figures below), thus generating two separate nanostructure’s configurations. In general, the DNN could not obtain good matching/results with the desired spectra, with the prediction of wrong nanostructures dimensions. It is quite expected as all the trained data of the GPN problem contained only configurations with a single nanostructure in one unit cell. While the spectra of two separate structures could seem for the naked eye similar to the spectra of a complex 2D nanostructure (like the case of two nanobars in different orientation vs, the spectra of a “L-shape”), the physics of both configuration is very different, mainly because in the single nanostructure, the free electrons are shared and can move freely in both orientation of the complex nanostructure, while in the two separate structures they cannot do so. Yet, there were two interesting families of predictions that are worth mentioning here: (i) the case of two parallel bars, and (ii) the case of one bar and one L-shape”.

(i)  In one case the structure is composed of two parallel nanobars, the DNN has been able to obtain very good results as can be seen in the Fig. S4 which is not the exact prediction, yet in fact a very similar solution that instead of separate legs with a length of 140 nm each, it predicts existence of one leg (#2) with 138 nm. Taking into account that each nanostructure is repeating itself periodically in an array configuration, it means that the network have been able to predict the best fitting configuration and obtain an accurate solution! but only with a different period length between the nanostructures, which influence on the density of the nanostructures in the array, thus on the deepness of the transmission at the resonance.


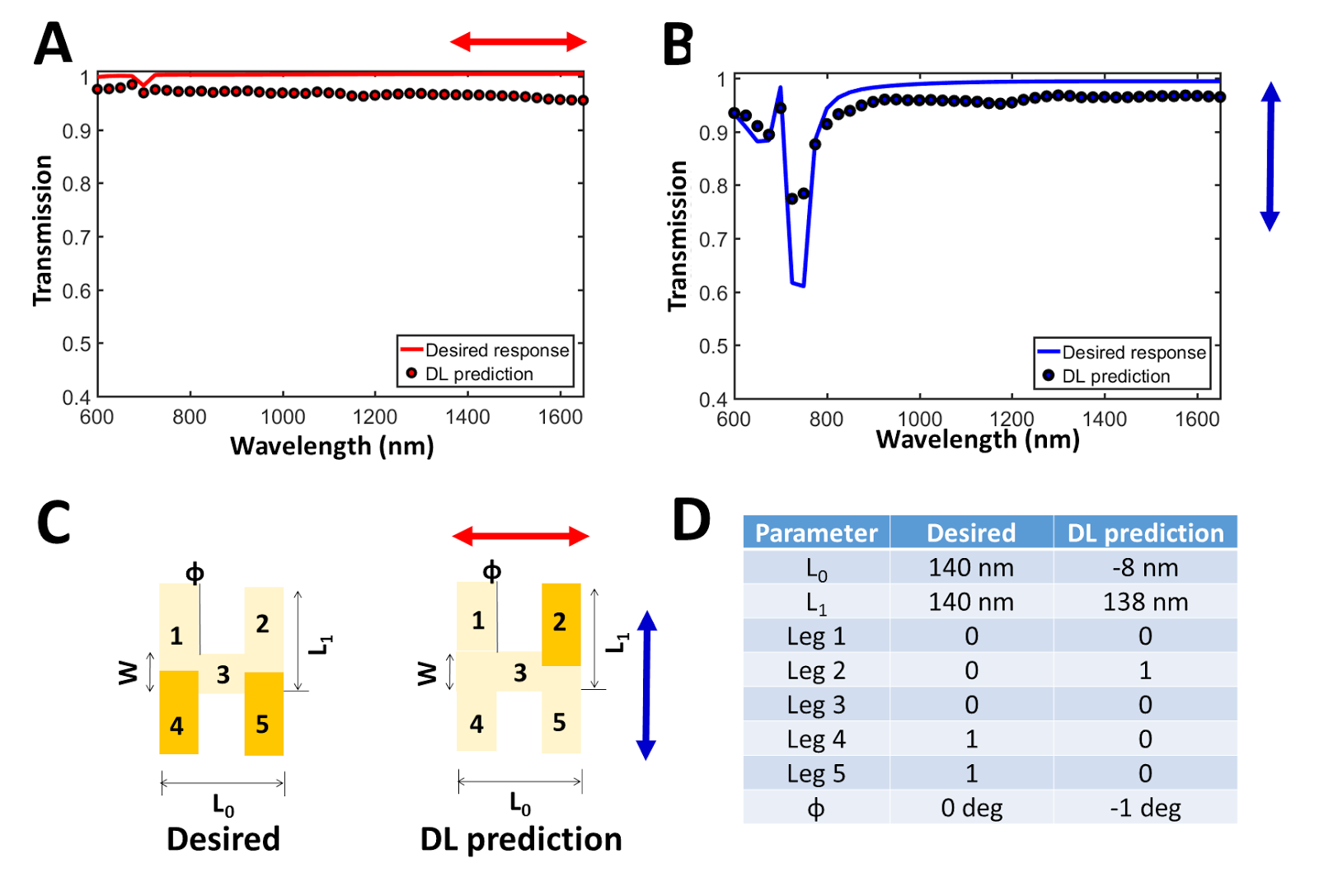


**(a)**

**(b)**

**(c)**

**(d)**

Figure S4 - Deep Learning retrieval of two parallel separate bars.

(ii)  In one case the structure is composed of one nanobar and one “L-shape”

The results are shown in the Fig. S5.


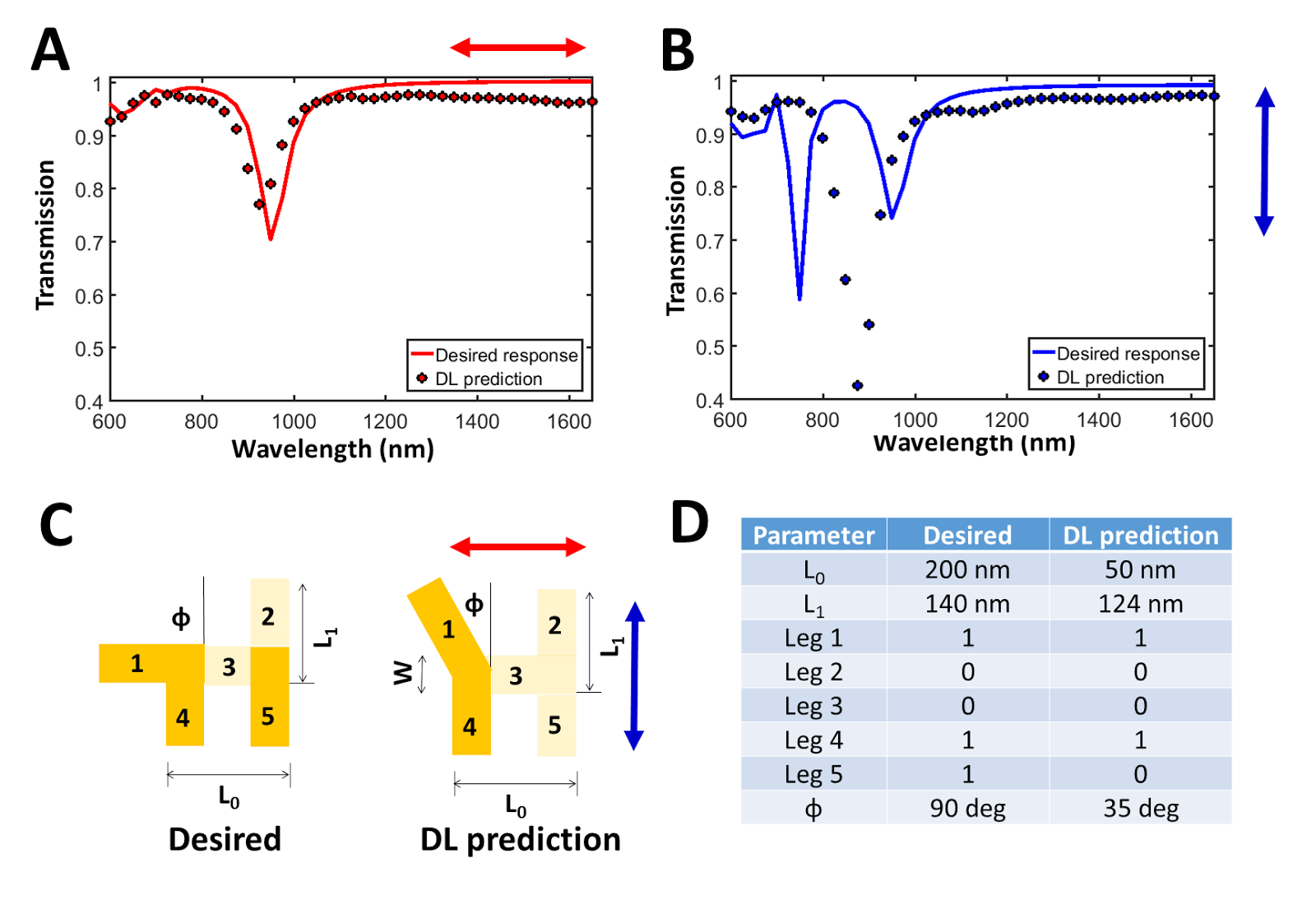


**(a)**

**(b)**

**(c)**

**(d)**

Fig. S5- Deep Learning retrieval of flipped L and a bar

As can be seen, the DNN could predict a nanostructure with an accurate spectral response only in the horizontal axis, and fails to accurately predict vertical spectral response. Yet, bearing in mind that the network had been training on a single configuration per unit cell which poses a constraint on finding the best fitting, the predicted geometry and lengths of the nanostructure by the DNN in this case is very appropriate: In the horizontal direction - the resonance should be proportional to the effective length at that direction, which is appropriate (taking into account the width of the nanostructure). In the vertical axis, for the two separate nanostructures there are two distinct resonance (each for nanostructure), but in the DL prediction, which is constrained (from its perspective) to finding the nanostructure that will give the average of both resonances. In some sense the predicted geometry can be thought as a superposition of the two single ones.

We stress again that in both cases, we have not included those geometries in the training set, and actually did not perform the Comsol simulations for these structures except for the analysis done in this section. We believe that with a proper training, a DNN can built an appropriate model that can include two (and even multiple) nanostructures in a unit cell.

1. Further information on the numerical COMSOL Simulation

We performed finite element method (FEM) simulations using the 'Electromagnetic Waves, Frequency Domain' module of the COMSOL 4.3b commercial software. For consistency, the edges of the nanostructures were made using fillets with the constant radius of 15 nm and the cross-section of the simulation was 700 nm in the X and Y directions. We have considered geometries based on a five edges shape of 'H' while varying an angle of one of the edges, the existing edges and the edges lengths.

**(b)**

**(a)**


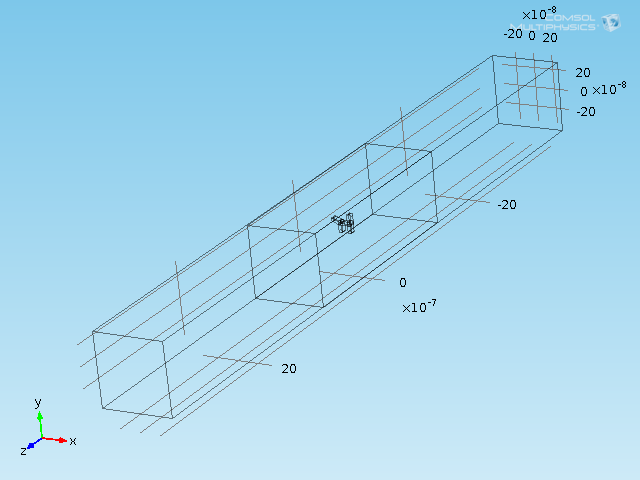

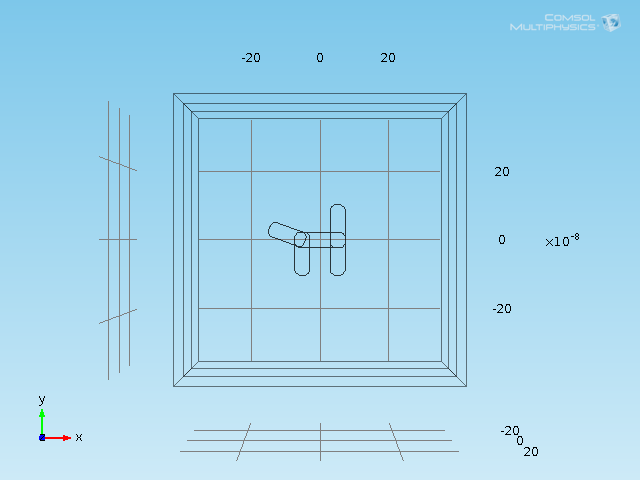


Fig. S6 (a) Comsols 'Graphics' of the simulation (b) Cross-section of the Comsols 'Graphics'

The nanostructure is simulated (Fig.S6) in a homogeneous dielectric medium with a chosen real effective-permittivity. For preventing reflections from the far planes, PMLs with a depth of the maximum wavelength were placed on both far ends of the homogeneous medium in the propagation direction of the radiating field. The remaining surfaces had 'Periodic Condition' boundary conditions to simulate a 2D array, as in the fabrication process. The transmission calculation was done by integrating the Poynting vector over the far surface and dividing by the transmission of the case without a geometry.

For the dataset predicting the fabrications, the nanostructure was made of Gold with a wavelength dependent homogeneous medium permittivity ($\epsilon_{d}\left( \lambda\right)$) such that $\epsilon_{d}(\lambda)=\frac{1}{2}(\epsilon_{ITO}\left( \lambda\right)+\epsilon_{AIR})$, where $\epsilon_{AIR}$ stands for the air permittivity and equals 1 and $\epsilon_{ITO}\left( \lambda\right)$ is the ITO permittivity which stands for the adhesion layer between the glass and the Gold nanostructure. $\epsilon_{ITO}\left( \lambda\right)$ is wavelength dependent such that its imaginary part can be neglected in the measured spectrum range. Unfortunately, when metallic inclusions are placed in the medium the effective permittivity of the medium doesn't have a closed analytical form [1]. We found that the approximation for the effective permittivity of the medium fitted the measurements for structures with resonances at the high wavelength range, probably because the difference between $\epsilon_{ITO}\left( \lambda\right)$ and $\epsilon_{AIR}$ was about 1.5 or lower at that range.

A justification for ignoring the glass permittivity can be found in [2] and [3]. In [2] it was shown that changes in the thickness of a Titanium adhesion layer higher than 40% of the nanostructures height, doesn't affect the plasmon resonance. In [3] it was shown that for an Au nanoparticle with diameter of 10 nm and a graphene layer, the LSPR shifting saturates when the distance between the nanoparticle and the graphene layer is more than 20 nm. A prediction for a similar behavior of the ITO layer is assumed. In our case, the ITO thickness is about 100 nm which is about 250% of the nanostructure thickness of about 40 nm.

For the sake of completeness, we have performed a satisfactory comparison of the prediction performance between the DNN trained on the ITO-enabled dataset and the DNN trained without taking into account the ITO layer.

References

1. M. G Silveirinha, C. A. Fernandes, “Effective permittivity of a medium with stratified dielectric host and metallic inclusions”, IEEE AP-S International Symp. 4, 3777–3780 (2004).
2. B. Lahiri, R. Dylewicz, R. M. De La Rue, N. P. Johnson, "Impact of titanium adhesion layers on the response of arrays of metallic split-ring resonators (SRRs)," Opt. Express 18, 11202-11208 (2010).
3. J. Niu, Y. Jun Shin, J. Son, Youngbin Lee, J-H. Ahn, H. Yang, "Shifting of surface plasmon resonance due to electromagnetic coupling between graphene and Au nanoparticles," Opt. Express 20, 19690-19696 (2012).
